# Determination of insoluble calcium content in Cheddar, feta, Juustoleipa, and mozzarella cheeses using acid-base buffering curves

**DOI:** 10.3168/jdsc.2024-0561

**Published:** 2024-07-20

**Authors:** Rachel Lindstrom, Prateek Sharma

**Affiliations:** Nutrition, Dietetics and Food Sciences Department, Utah State University, Logan, UT 84322

## Abstract

The amount of colloidal calcium phosphate (CCP) complex associated with caseins (insoluble [INSOL] Ca) determines the body, texture, flavor, and breakdown of cheese constituents during aging. The continuous pH decline during cheesemaking because of lactic acid fermentation results in solubilization of INSOL Ca. Measuring INSOL Ca in such a dynamic and wide range pH system (6.6 to 5.3) is challenging. The purpose of this study was to test utility of an acid-base auto-titration method in differentiating INSOL Ca content in cheeses with a wide range of pH (i.e., Juustoleipa 6.6, mozzarella 5.6, Cheddar 5.3, feta 4.8) and also to understand the relationship between pH, protein content, and INSOL Ca. A positive relationship was obtained for pH (R^2^ = 0.62) and protein content (R^2^ = 0.85) with INSOL Ca, suggesting concomitant release of CCP content at lower pH values and association of higher amount of CCP with higher protein content. Despite having a pH slightly closer to mozzarella (pH 5.56), Cheddar cheese (pH 5.25) had more INSOL Ca (0.67%) owing to the highest amount of protein (26%) and low moisture content (33%). Feta had the lowest amount of INSOL Ca (0.15%) owing to a low pH (4.79), higher moisture content (55%), and low protein content (14%). Juustoleipa had the highest percentage of INSOL Ca out of total Ca (88%) due to higher pH (6.62) and more intact casein. It was evident that the acid-base titration method was able to differentiate INSOL Ca between different types of cheeses with varying pH and protein content. Findings of this work will help tracking the proportion of INSOL Ca at various stages of cheesemaking and understanding kinetics of INSOL Ca solubilization and its role in causing pH variation in the early stage of cheese ripening.

## Highlights

• Cheese pH was positively correlated with the amount of INSOL calcium out of total calcium.
• Juustoleipa was found to have the most INSOL calcium, and feta had the lowest.
• Final pH of the cheese and extent of proteolysis affect the proportion of INSOL calcium.

## Graphical Abstract

**Figure:**
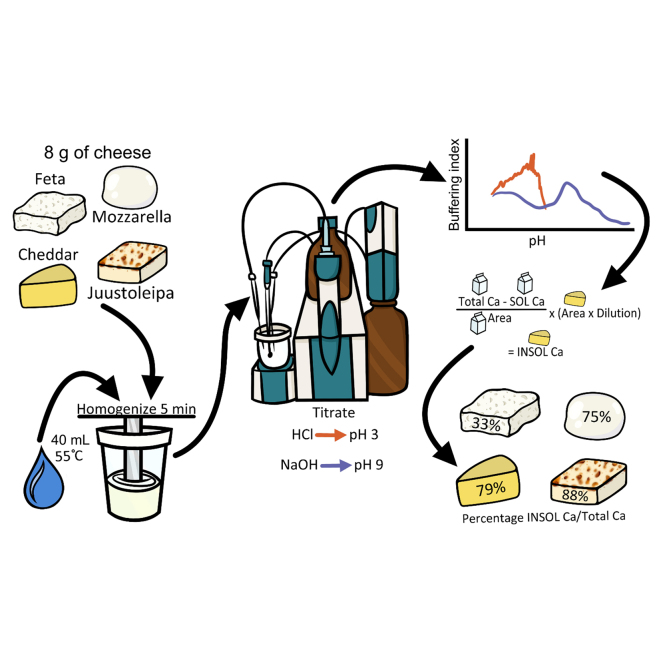
**Summary:** Cheese pH and amount of insoluble (INSOL) calcium content are critical factors in determining the physicochemical, textural, and functional properties of various cheese types. In this study, we wanted to evaluate whether the acid-base titration method can be used to differentiate the effect of cheese pH on INSOL calcium content. Four different cheese types (i.e., Cheddar, feta, Juustoleipa, and mozzarella) with varying pH (4.8-6.6) were successfully tested for INSOL calcium content using acid-base buffering curves. Cheeses with higher pH and protein content and less extent of proteolysis had higher INSOL calcium content. This clearly indicates that INSOL calcium content plays an important role in the cheese buffering effect. Overall, acid-base titration was very useful in differentiating between INSOL calcium content of cheeses with varying pH and extent of proteolysis. These findings can be used in understanding the kinetics of INSOL calcium release in the cheese matrix during the early stage of aging.

The United States is one of the largest consumers of cheese, having almost doubled per capita consumption in the last few years (Boisseau, 2020; Hutchins and Hurley, 2024). Cheddar cheese is the second most consumed cheese in the United States after mozzarella (Pace et al., 2024; USDA, 2024). Mozzarella is a soft or semi-soft Pasta Filata type cheese originated from Italy. It is produced by reducing pH either due to starter culture addition or direct acidification, followed by kneading-stretching of the curd either by hand or mechanical mixers or workers to give a fibrous appearance to the cheese texture (Sharma et al., 2017). Mozzarella cheese is widely used as pizza topping with expectation of having optimum stretching, melting, free-oil formation, browning, and blistering characteristics (Rowney et al., 1999; McSweeney et al., 2004; Jana and Mandal, 2011).

Cheddar cheese is a hard or semihard variety of cheese originated from England. Cheddar cheese is made by the cheddaring process wherein the curds are pressed together, then stacked and turned frequently until pH reaches approximately 5.2. The curds are then salted and pressed before they reach their final pH of 5.1 to 5.3 and moisture content around 37% (McSweeney et al., 2004).

Feta is a pickled soft Greek style cheese, traditionally made from goat or sheep milk. Feta has a typical “baby burp”-like flavor that comes from the lipase activity. The final pH of this cheese ranges from 4.6 to 5.3 during the storage in brine until further use (McSweeney et al., 2004; McMahon et al., 2009; Hammam et al., 2021).

Juustoleipa is a hard “bread cheese” similar to paneer and Halloumi, originated from northern Finland and Sweden for over 200 years. One unique property of this cheese is that it does not melt upon heating. This is partly due to the addition of Ca chloride when the cheese is being made and the high pH (6.1–6.6) of the cheese (Zady et al., 2019).

Due to the wide pH range of the 4 experimental cheeses (mozzarella, Cheddar, feta, and Juustoleipa), differences in the amount the Ca associated with rennetted casein particles present in the cheese matrices are expected (Lucey et al., 1993a; Hassan et al., 2004). These differences could also lead to diversity in the material characteristics due to changes in the cheese ripening profile (i.e., rate of lactic acid production from lactose breakdown, body and texture, flavor profile of the cheeses). For example, Juustoleipa has a higher pH (∼6.2) with minimum extent of proteolysis causing a rubbery texture, whereas feta has a much lower pH exhibiting crumbly texture (Lucey and Fox, 1993). Many factors affect the final pH of a cheese such as activity of starter cultures, rate of proteolysis, and Ca content associated with proteins (Hassan et al., 2004; McSweeney, 2017). It is well known fact that drop in the pH during cheese ripening leads to solubilization of colloidal calcium phosphate (**CCP**) complex associated with caseins. However, it is not yet fully understood how different quantities of Ca associated with caseins would affect the final pH of the cheese either due to the buffering effect of residual phosphate ions or manipulation of proteolytic behavior. This study is focused on understanding the role of insoluble (**INSOL**) form of Ca phosphate on the final pH of these diverse cheeses.

Within the dairy matrices, Ca is found in several different soluble (**SOL**) and INSOL forms (Fox et al., 2016). Within the INSOL Ca, CCP is associated with casein micellar structure in the liquid milk. The CCP acts as a buffering agent in protein-rich dairy products such as cheese, due to release of phosphate ion combining with H^+^ ions resisting pH change upon acidification (Lucey et al., 1993b; Upreti et al., 2006). During cheesemaking after rennet coagulation, at higher pH the INSOL Ca phosphate (in the form of CCP) stays with the partially dissociated casein micelles holding cheese coagulum structure together (Hassan et al., 2004). During cheesemaking the state of Ca changes from the INSOL form to SOL form or vice versa depending upon the shift in pH and temperature of the cheese (Mizuno et al., 2009; Fox et al., 2015). Because of Ca mobility between colloidal and SOL phases, accurate measurement of INSOL Ca during cheesemaking is challenging. Our laboratory is working on developing a protocol for preserving the state of Ca during sample collection, handling and testing so that accurate quantity of INSOL Ca can be determined at specific step of cheesemaking. Two methods are reported in the literature to measure INSOL Ca, the cheese juice method and acid-base titration (buffering) method (Hassan et al., 2004; Lamichhane et al., 2019). Researchers have also used Fourier transform infrared spectroscopy for measuring bound Ca and organic phosphorus (Upreti and Metzger, 2006). The acid-base titration method has traditionally been used for determining buffering capacity of various dairy products (Lucey et al., 1993a,b; Salaün et al., 2005); however, its use for determining quantity of INSOL Ca in cheese was not established until Hassan et al. (2004) developed a method and validated it with the cheese juice method. The acid-base titration method has now successfully been used for accurately measuring INSOL Ca in the Cheddar cheese (Hassan et al., 2004; O'Mahony et al., 2005, 2006), mozzarella (Mizuno et al., 2009), and feta (Remillard and Britten, 2011). However, the relationship between pH and extent of proteolysis of the different cheese varieties and INSOL Ca content has not yet been reported. The purpose of this study was to test the applicability of acid-base titration method developed by Hassan et al. (2004) on cheeses with varying the position, pH, and INSOL content and understanding the relationship between these factors. Due to the wide pH range of the experimental cheeses, they were expected to have varying content of total Ca and CCP (INSOL Ca) and their association with protein matrix and aqueous phase. Our hypothesis is that if this method is able to differentiate between INSOL Ca content in cheese prepared at different pH, this protocol can be used to capture proportion of INSOL and SOL Ca during cheesemaking (pH ranges from 6.6 to 5.35) and understand the dynamics of INSOL Ca mobility during subsequent processing and storage.

All the cheeses were produced in the Gary Haight Richardson Dairy Products Laboratory at Utah State University (Logan, UT) from the whole milk obtained from the sole source, the George B. Caine Dairy Teaching and Research Center (Wellsville, UT). The whole milk was pasteurized at 73°C for 15 s using HTST pasteurizer. The feta, Juustoleipa, and mozzarella were produced on the same day (March 8, 2023) with the same milk. Each of these cheeses (except Cheddar) was made in an open vat with 136 kg of milk. The Cheddar was produced in less than a week (March 14, 2023) using a Tetra Scherping horizontal cheese vat (Tetra Pak Cheese and Powder Systems Inc., Winsted, MN) with 680 kg of milk. Milk was preserved from each of the cheese make days for the SOL Ca analysis.

The Cheddar cheese was made using a protocol from McMahon et al. (2014, 2022). Feta was prepared using McMahon et al. (2009) with a few modifications. The amount of starter culture added was adjusted to 5 g of starter culture (Choozit Feta FT001, Danisco, Denmark) with 1 g of adjunct culture (Danisco Holdbac LC, Denmark). Two types of lipases were used: 9 g of Danisco kid lipase 300 and 8 g of Danisco calf lipase 600. The mozzarella was produced using the direct acidification method from McMahon et al. (2005). The milk was acidified to pH 5.60 using 5% vinegar (Food Club from Walmart, Logan, UT) and renneted using double-strength chymosin (Maxiren, US, ∼650 international milk clotting units/mL; DSM Food Specialties USA Inc.). After cutting, whey drainage, and salting, the cheese curd was stretched in the hot brine solution (70°C) followed by cooling to 5°C and packing in the vacuum-sealed bag.

For the Juustoleipa cheese, 0.04% of Ca chloride (32%) solution (Nelson and Jameson, Marshfield, WI) was added to the milk followed by warming up to 37°C before renneting. After a 30-min setting period the cheese was cut into 1.6-cm cubes using a wire cutter and stirred slowly for over 35 min, followed by whey drainage at pH 6.55. The NaCl at 0.33% of the cheese curd was added in 2 applications. The cheese was hooped in 22-lb (∼10 kg) blocks and pressed at 40 psi (2.76 bar) for 90 min. Pressed blocks were sliced into 3/4 inch (1.9 cm) thick disks and baked using a pizza oven for 6 min at 285°C oven temperature with the aim of achieving an internal cheese temperature of 82°C. Baked cheeses were vacuum sealed and cooled. All the samples were stored at 4°C until their use. All testing of the cheese samples was performed within 3 mo of their manufacture.

The cheese composition was analyzed using standard analytical methods. The total Ca was measured using inductively coupled plasma optical emission spectroscopy (**ICP-OES**) by the analytical laboratory at Utah State University following the protocol from Panthi et al. (2019). The pH of each cheese was measured using an Orion Star A11 pH meter (Vernon Hills, IL). The moisture of the cheese was measured using a CEM Turbo Rapid Moisture Analyzer CEM (CEM, Matthews, NC). The protein was measured using CEM Sprint Rapid Protein Analyzer (CEM). Urea-PAGE was performed using the method described by Pace et al. (2024) to understand the casein breakdown within each of the cheeses.

The SOL Ca of the milk was measured by ultracentrifugation of milk at 100,000 × *g* for 1 h at 25°C (Lin et al., 2017). The supernatant was then drawn from the middle portion of the clear aqueous phase using a pipette and was analyzed for SOL Ca by the analytical laboratory at Utah State University using ICP-OES technique. The INSOL Ca in the cheese milk was obtained after subtracting SOL Ca from the total Ca.

The INSOL Ca in cheese was measured using the method developed by Hassan et al. (2004) with some modifications. Briefly, 8 g of grated cheese was homogenized into a cheese slurry with 40 mL of 55°C distilled water and then allowed to cool to room temperature (22°C). An 888 Titrando using a 6.0256.100 pH meter probe (Metrohm USA, Riverview, FL) and the Tiamo 2.5 software were used to auto-titrate the samples. The cheese slurry was titrated with 0.5 *M* HCl from initial pH to a pH of 3, followed by back titration with 0.5 *M* NaOH to a pH of 9. Titrant was added to the cheese slurry slowly at 0.1 mL of solution at a time. Cumulative volume of acid or base added and instant pH were recorded by the Titrando software. The buffering index (dB/dpH) of cheese and milk samples was calculated using the equation reported by Hassan et al. (2004):

[1] $$\frac{dB}{dpH}=\;\frac{mLofacidorbaseadded\times normalityofacidorbase}{volumeofsample\times pHchangeproduced}.$$

The buffering index was then plotted on a graph by keeping pH on the x-axis and buffering index on the y-axis (Figure 1). The area between the forward and backward titration within certain pH range (near the buffering peak) was used as an indicator for the quantity of INSOL Ca.

[2] $$\begin{array}{l} INSOLCainmilk\left(\frac{mg}{100g}\right)=\\ totalCainmilk\left(\frac{mg}{100g}\right)-SOLCainthemilk\left(\frac{mg}{100g}\right) \end{array}$$

[3] $$\begin{array}{l} INSOLCacontent\left(\frac{mg}{100g}\right)=\\ \;\frac{INSOL\;Ca\;in\;milk\left(\frac{mg}{100g}\right)}{A_{M}}\times \left(A_{C}\times D\right) \end{array}$$

[4] $$\begin{array}{l} INSOLCaincheese\left(\%\right)=\;\\ \frac{INSOLCacontent\left(\frac{mg}{100g}\right)}{totalCacontentofcheese\left(\frac{mg}{100g}\right)}\times 100\% \end{array}$$

In the equations *A_M_* and *A_C_* represent the area of the milk and the area of the cheese, respectively. *D* is the dilution factor.

**Figure 1:**
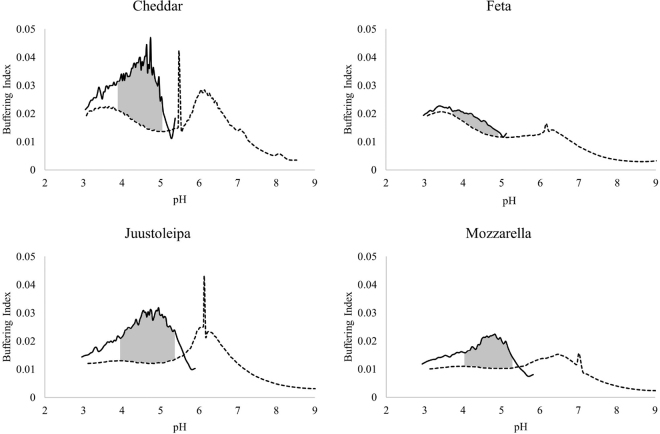
Acid-base buffering curves of Cheddar, feta, Juustoleipa, and mozzarella. Forward titrated to pH of 3 with 0.5 *M* HCl, then back titrated to pH of 9 with 0.5 *M* NaOH. **—** Forward titration; — back titration; the gray shaded area indicates the approximate area used to calculate INSOL Ca.

The pH ranges for calculating differential area were different for each cheese based upon the point where both curves tend to be parallel to each other: for Cheddar 4.0 to 5.1, similar to Hassan et al. (2004); feta 4.8 to 3.4; Juustoleipa 5.2 to 3.8; and mozzarella 5.3 to 4.1. As per the equations above (Equations 1–4), the cheese milk was used for calibrating the area between forward and backward buffering curves (pH range 4.1–5.8) to get INSOL Ca.

All testing on the samples was performed in triplicate. The statistical analysis for comparing the composition and INSOL Ca of the different cheese types was performed using IBM SPSS Statistics package (version 27, IBM SPSS Inc.). One-way ANOVA with post hoc Tukey's test 5% significance level was used for comparing means between different samples.

The composition of the Cheddar, feta, and Juustoleipa cheese is presented in the Table 1. The Cheddar had a lower moisture content (33%) than other cheeses. This was expected as Cheddar is a slightly low moisture variety (Bradley and Vanderwarn, 2001) compared with the feta and Juustoleipa (Jalili, 2016; Zady et al., 2019). However, the Cheddar used in this study had less moisture content than the Cheddar cheese reported in the literature (Bradley and Vanderwarn, 2001; Hassan et al., 2004), which can be attributed to the differences in the procedure used particularly in relation to the cut size of the curd and duration of pressing of milled and salted curd (McMahon et al., 2022). Cheddar contained the highest amount of protein among all cheeses, which could be attributed to the low moisture content in the cheese (Table 1). Cheddar (0.86%) had the highest total Ca content followed by Juustoleipa (0.76%), feta (0.55%), and mozzarella (0.46%), which could be attributed to variations in the final pH of the experimental cheeses (Table 1). At higher pH (particularly drain pH) more Ca is retained in the curd protein matrix in the form of CCP (Tunick et al., 2007). Juustoleipa had a slightly lower Ca content than reported in the literature (Zady et al., 2019), which could be attributed to the difference in the amount of Ca added or retention in the curd due to variations in make procedures. Mozzarella (50%) had the second highest (*P* < 0.05) moisture content after feta (55%); however, it had the second lowest percentage of total Ca owing to low pH (4.79) causing extensive solubilization of CCP and leaching to the storing brine solution.

**Table 1:** Composition of the 4 cheeses used for testing suitability of the acid-base buffering method for determining INSOL Ca^1^

Composition	Cheddar	Feta	Juustoleipa	Mozzarella
Protein (%)	26.0 ± 1.1^a^	14.0 ± 0.4^b^	23.0 ± 0.8^c^	22.0 ± 1.1^c^
Moisture (%)	33.0 ± 0.7^a^	55.0 ± 1.1^b^	42.0 ± 1.0^c^	50.0 ± 0.5^d^
Fat (%)	36.0 ± 0.3^a^	23.0 ± 0.3^b^	26.0 ± 0.3^c^	25.0 ± 0.0^d^
pH	5.25 ± 0.19^a^	4.79 ± 0.05^b^	6.62 ± 0.18^c^	5.56 ± 0.02^d^
Na (%)	0.66 ± 0.15^a^	1.08 ± 0.11^b^	0.33 ± 0.01^c^	0.50 ± 0.03^ac^
P (%)	0.44 ± 0.12^ab^	0.30 ± 0.01^a^	0.47 ± 0.03^b^	0.39 ± 0.01^ab^
Total Ca (%)	0.86 ± 0.03^a^	0.46 ± 0.00^b^	0.76 ± 0.06^c^	0.55 ± 0.02^d^
INSOL Ca (%)	0.67 ± 0.02^a^	0.15 ± 0.02^b^	0.67 ± 0.03^c^	0.41 ± 0.01^d^
INSOL Ca of total Ca (%)	79 ± 3^a^	33 ± 4^b^	88 ± 7^c^	75 ± 4^ad^

a–d Different superscripts in a column indicate a significant difference at α = 0.5.

1 Data represent mean ± SD.

The acid-base buffering curves of Cheddar, feta, Juustoleipa, and mozzarella are shown in Figure 1. When the cheeses were forward titrated (initial pH to pH 3.0) with 0.5 *M* HCl, a peak was observed, indicating a buffering effect due to the solubilization of CCP from the protein phase, which ultimately dissociates into ions and causes the release of phosphate and Ca ions interacting with counter ions present in the aqueous phase (Lucey and Fox, 1993; Hassan et al., 2004; Remillard and Britten, 2011). The acid buffering peak was observed at pH ∼4.8 for Cheddar cheese, similar to the peaks reported by Hassan et al. (2004); however, these peaks were at the lower pH compared with the buffering peak (pH 5.1) for the milk (Remillard and Britten, 2011). This can be linked with the first pKa of the phosphate groups and precipitation of multiple forms of Ca salts in the cheese matrix (Remillard and Britten, 2011). Differences in the buffering peak for milk and cheese samples could also be related to the solid behavior of cheese causing slow penetration of acid into the structure (Hassan et al., 2004). The forward buffering peaks (during acidification) of Juustoleipa and mozzarella were at pH ∼4.9 and pH ∼4.8, respectively, which were close to Cheddar cheese (pH 4.8). This could indicate that irrespective of differences in the cheese types the maximum buffering effect of residual CCP occurs around 4.8 (Hassan et al., 2004). Feta exhibited a distinctive buffering behavior with the absence of a definite peak around 4.8, which could be attributed to the low pH 4.78 (Table 1). At this low pH, most of the CCP has already been solubilized even before the titration and therefore loses its ability to exert any buffering effect.

While back titrating (lower to higher pH) cheeses with 0.5 *M* NaOH, no peak was observed around 4.8, indicating no buffering at this pH possibly due to presence of the dissociated Ca phosphate salts. The back titration peak for Cheddar, feta, and Juustoleipa was observed at pH ∼6.2, which was similar to the trend reported by Hassan et al. (2004). This second peak can be associated with the formation of Ca phosphate (Lucey et al., 1993b) near second pKa of the phosphate groups at which another buffering peak is normally observed.

The shaded area between forward and backward titration (Figure 1) curves relates to the quantity of INSOL Ca in the cheese matrix. It is proportional to the amount of CCP solubilized during acidification. Irrespective of pH differences of Cheddar (pH 5.25) and Juustoleipa (pH 6.62), both cheeses exhibited similar differential area under the curve indicating the same absolute quantity of INSOL Ca (0.67% Table 1; Figure 1). However, the proportion of INSOL Ca over total Ca in the cheese matrix was higher (*P* < 0.05) for Juustoleipa (88%) than for Cheddar (79%; Table 1). No previous data are available on INSOL Ca content in the Juustoleipa cheese; however, higher retention of INSOL Ca is most likely due to the higher pH of the Juustoleipa cheese (6.62). Further, during baking Juustoleipa at higher pH would form a stronger network due to increased hydrophobic interactions between renneted casein in the presence of extra Ca content, leading to more retention of INSOL Ca in the curd matrix (Sharma et al., 2016). A higher proportion of INSOL Ca content within the cheese matrix causes increased hardness and reduces melting characteristics, which are desirable properties in Juustoleipa cheese (Zady et al., 2019). The proportion of INSOL Ca content reported in our study for Cheddar cheese (79%) confirms previous observations using titration method ranging from 65% to 76% (Hassan et al., 2004; O'Mahony et al., 2005). The slightly higher value of INSOL Ca in our study can be attributed to higher cheese pH (5.25). At higher pH, formation of more INSOL Ca is expected due to poor solubility of CCP (Hassan et al., 2004). Moreover, final INSOL content in cheese would depend upon composition of cheese milk, starter culture, cheese manufacturing process, and storage temperature.

Mozzarella cheese had the next highest percentage of INSOL Ca (0.41%, Table 1). The mozzarella percentage of INSOL Ca to total Ca (75%) was comparable (using titration method) to that which is found in literature at drain pH of 6.25 (Mizuno et al., 2009). Lower drain pH mozzarella cheese is reported to have lower amount of INSOL Ca causing a decrease in hardness and increasing meltability due to a decrease in the INSOL Ca (Mizuno et al., 2009). The shaded area for feta was extremely small, and therefore had only 0.15% of INSOL Ca and only 33% of the total Ca was INSOL Ca. These low numbers of INSOL Ca indicate that due to low pH, feta cheese lost most of the CCP during the cheesemaking process.

When comparing all of the cheeses, a positive correlation was obtained between pH and INSOL Ca, suggesting the release of CCP content with pH decrease (Figure 2a). Higher pH leads to more retention of CCP within the curd matrix either due to increased Ca induced interactions between caseins (Sharma et al., 2016) or loss of solubility of CCP complex, causing shift in the equilibrium. A positive correlation was obtained between protein content and the amount of INSOL Ca present (Figure 2b), indicating that higher levels of CCP are associated with higher protein content in the curd matrix, forming a basis for extensive protein-protein interactions. Solubilization of INSOL Ca phosphate due to low pH or varying amount of intact casein can also cause variations in the CCP content of cheese (Remillard and Britten, 2011).

**Figure 2:**
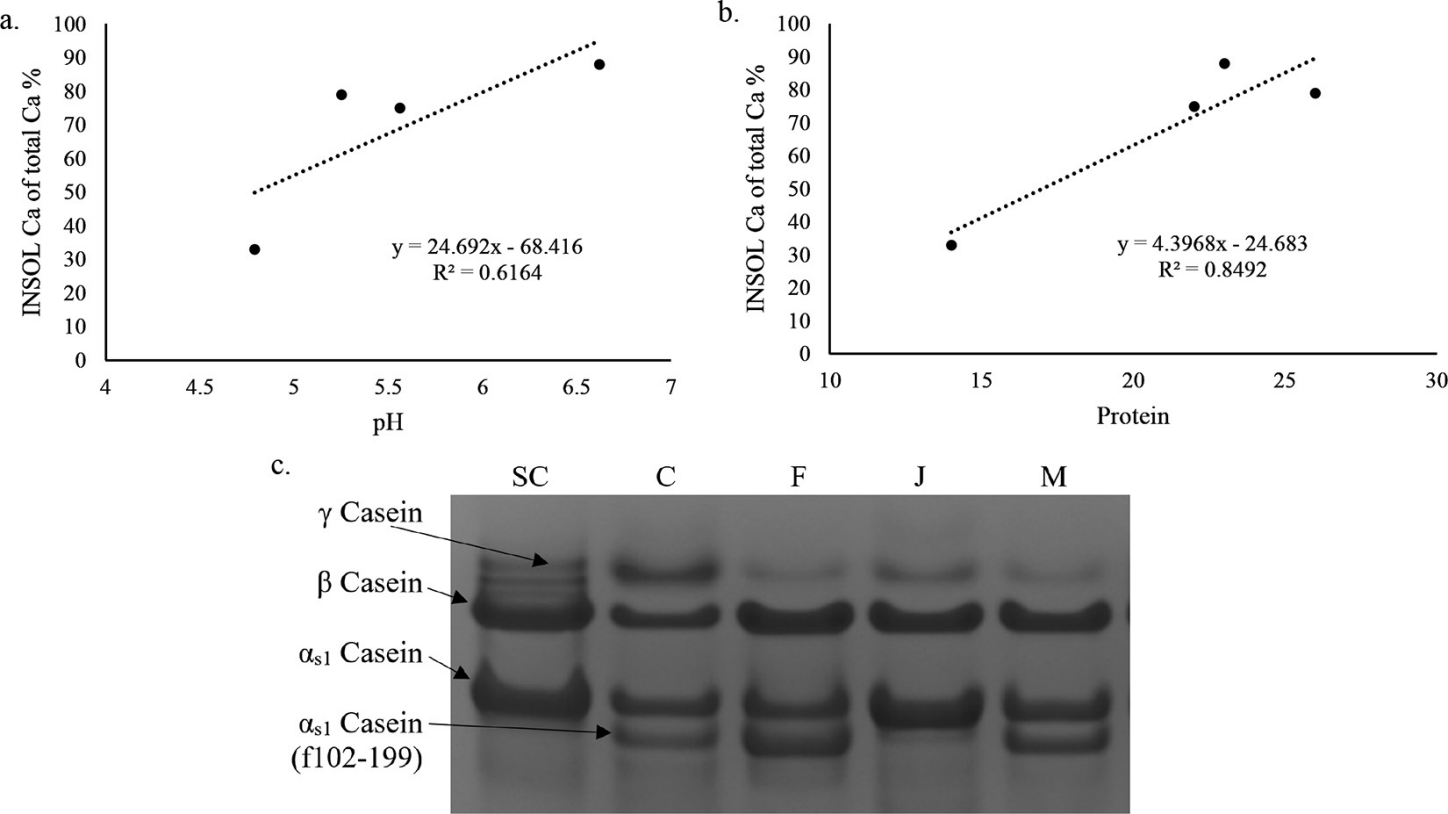
Relationships between % INSOL Ca of total Ca and pH (a), and between % INSOL Ca of total Ca and protein (b) in 4 different types of cheeses; and electrophoretic pattern of various cheese proteins on urea-PAGE with the casein proteins identified (c). SC = sodium caseinate; C = Cheddar; M = mozzarella; F = feta; J = Juustoleipa.

A breakdown pattern of caseins (β- and α_S1_-casein) due to rennet action and proteolytic activity of starter cultures is shown in the urea-PAGE images (Figure 2c). Juustoleipa exhibited thick intact β-casein and α_S1_-casein bands very similar to the one shown for the sodium caseinate and absence of smaller fractions of caseins, indicating that most of the caseins were intact in this cheese type. This also indicates less extent of primary and secondary proteolysis of caseins in Juustoleipa possibly due to inactivation of residual rennet activity upon baking. However, Cheddar, feta, and mozzarella exhibited an extra band below α_S1_-casein, suggesting the release of an α_S1_-casein fragment (f102–199) because of renneting action (Lamichhane et al., 2019). The appearance of these fragments on an electrophoretic pattern indicates the occurrence of some extent of proteolysis (in less than 3 mo of storage) within the cheese matrix. The α_S1_-casein (f102–199) band for feta cheese appears to be slightly darker than mozzarella and Cheddar, which is an indication of more proteolysis taking place within the feta cheese during manufacture and less than 3 mo of storage. Low pH cheese (e.g., feta) had a lower quantity of INSOL Ca (0.15%), holding the casein network loosely due to increased hydration, therefore increasing susceptibility of caseins to proteolytic enzymes (O'Mahony et al., 2005). In contrast, the presence of a higher level of intact caseins indicates higher proportions of INSOL Ca within the cheese matrix holding the protein network tightly (O'Mahony et al., 2006). Slower breakdown of the proteins is expected to cause slow release of CCP into aqueous phase. This explains that not only pH but also the extent of proteolysis can affect the INSOL Ca content in the cheese. Therefore, Juustoleipa (88%) had higher proportion of INSOL Ca compared with feta (33%).

Overall, acid-base titration was useful in differentiating between INSOL Ca content of cheeses with varying pH and extent of proteolysis. The protein content was positively related to the INSOL Ca content, suggesting that higher protein content in the cheese would lead to higher amount of INSOL Ca, and therefore more buffering effect. Higher levels of INSOL Ca were associated with cheeses made at higher pH indicating a possible role of INSOL Ca on the evolution of final pH of cheese. With an understanding of how different cheese types with varying Ca content and manufacturing conditions would affect the state of CCP, exact causes of pH variations in the early state of cheese ripening can be identified and controlled. Therefore, we intend to apply this knowledge in monitoring levels of INSOL Ca throughout the Cheddar cheesemaking process and understanding the role of kinetics of CCP and INSOL Ca migration on the evolution of pH in the early stages of ripening and its overall impact on cheese quality.
